# Modification of *N^6^*-methyladenosine RNA methylation on heat shock protein expression

**DOI:** 10.1371/journal.pone.0198604

**Published:** 2018-06-14

**Authors:** Jiayao Yu, Yi Li, Tian Wang, Xiang Zhong

**Affiliations:** College of Animal Science and Technology, Nanjing Agricultural University, Nanjing, Jiangsu, People’s Republic of China; Technische Universitat Munchen, GERMANY

## Abstract

This study was conducted to investigate effect of *N*^6^-methyladenosine (m^6^A) RNA methylation on Heat shock proteins (HSPs) and dissect the profile of HSP RNA methylation. The results showed that m^6^A methyltransferases *METTL3* mRNA was decreased in responses to heat shock stress in HepG2 cells, but m^6^A-specific binding protein YTHDF2 mRNA was upregulated in a manner similar to HSP70 induction. Immunofluorescence staining showed that the majority of YTHDF2 was present in the cytosol, however, nearly all YTHDF2 translocated from the cytosol into the nucleus after heat shock. METTL3 knockdown significantly changed *HSP70*, *HSP60*, and *HSP27* mRNA expression in HepG2 cells using siRNA, however, mRNA lifetime was not impacted. Silence of YTHDF2 using siRNA did not change expression of *HSP70*, but significantly increased *HSP90*, *HSP60*, and *HSPB1* mRNA expression. In addition, m^6^A-seq revealed that HSP m^6^A methylation peaks are mainly enriched on exons and around stop codons, and shows a unique distribution profile in the 5’UTR and 3’UTR. Knockdown of METTL3 changed the methylation patterns of HSPs transcript. In conclusion, m^6^A RNA methylation regulates HSP gene expression. Differential expression of HSPs modulated by m^6^A may depend on the m^6^A site and abundance of the target gene. This finding provides insights into new regulatory mechanisms of HSPs in normal and stress situations.

## Introduction

Heat shock proteins (HSPs), which are expressed constitutively in all species, are molecular chaperones that regulate protein localization, accumulation, and degradation, assist in protein re-folding, prevent protein aggregation [1], and play important physiological roles in normal conditions as well as situations involving both systemic and cellular stress [2, 3]. Based on their molecular sizes, HSP proteins are classified into a number of conserved protein families, including HSP90, HSP70, HSP60 and small HSPs [4]. It is well known that increase of HSP genes are activated at the transcriptional level by heat shock transcription factor 1 (HSF1) [5]. Interestingly, growing evidence indicates that the expression of HSPs can be attributed to epigenetic changes including DNA methylation and acetylation of histones [6–9]. However, modulation of HSPs expression by RNA methylation remains unclear.

To date more than 100 diverse chemical modifications of RNA have been identified. *N*^6^-methyladenosine (m^6^A) is the most abundant internal modification of eukaryotic mRNA, influencing metabolism and function of mRNAs [10]. M^6^A RNA modifications are dynamically and reversibly regulated by methyltransferases including METTL3 and METTL14 [11], and demethylases including ALKBH5 and FTO [12]. Their functions are exerted by direct recognition by m^6^A-specific binding proteins including YTHDF1 and YTHDF2 [13, 14]. M^6^A shows the most enrichment in translation start sites, stop codons, and 3’UTRs [15, 16], suggesting that it plays important roles in mRNA biology. Indeed, modification of m^6^A on mRNA functionally regulates mRNA splicing [15, 17], export [18], localization, translation [19], and stability [13], suggesting that m^6^A RNA methylation is an epitranscriptomic mechanism that regulates gene expression.

The precise sites and abundance of m^6^A are highly regulated under normal conditions, however, previous reports showed that cellular stress, such as heat shock or ultraviolet radiation, alters characteristic distribution and function of m^6^A [15, 20]. This finding suggests that changes of m^6^A may contribute to tuning a number of genes expressed under stress conditions. Indeed, Zhou et al. [21] found that m^6^A modification altered by heat shock stress and activate Hsp70 mRNA translation. However, manipulation of m^6^A RNA methylation on other stress-inducible chaperones such as HSP90, HSP60, and HSP27 remain unknown.

Here, we investigated the topology and function of m^6^A modifications of HSPs RNA, including *HSPA1B*, *HSPA9*, *HSP90AA1*, *HSPD1*, *HSF1*, and HSPB1 using m^6^A-seq in HepG2 cells.

## Materials and methods

All the procedures were approved by the Institutional Animal Care and Use Committee of Nanjing Agricultural University, China.

### Cell lines

The human HepG2 cell line used in this study was obtained from the American Type Culture Collection (ATCC) at passage 5 and grown in DMEM (Gibco, Grand Island, NY, USA) media supplemented with 10% FBS, and 1% 100× Pen Strep. HepG2 cells were incubated with 5% CO_2_ and 95% air at 37°C.

### Heat shock

HepG2 cells were subjected to 41°C heat shock in a water bath for 1 h and then recovery incubation at 37°C The cells were harvested at 6 h, 12 h, 24 h after heat shock and processed for RT-PCR analysis.

### siRNA knockdown

Human *METTL3* and *YTHDF2* siRNAs were ordered from Qiagen (Hs-METTL3 with target sequence CTGCAAGTATGTTCACTATGA, Hs-YTHDF2 with target sequence AAGGACGTTCCCAATAGCCAA). Control siRNA is from Qiagen (1027281). Each siRNA was transfected into HepG2 cells using Lipofectamine RNAiMAX (Invitrogen) for siRNA following the manufacture’s protocols. At 48 h after the transfection, cells were harvested and stored at -80°C for further studies.

### RNA isolation and purification

Total RNA was isolated from HepG2 cells using TRIzol (Invitrogen), and contaminant DNA was removed using DNaseI. The RNA integrity was determined on 1% agarose gel with ethidium bromide staining. The concentrations of RNA were quantified by absorbance at 260 nm and 280 nm using a NanoDrop ND-1000 UV spectrophotometer. Messenger RNA for LC-MS/MS was extracted using PolyATtract^®^ mRNA Isolation System III (Promega) followed by further removal of contaminated rRNA by using the Ribominus eukaryote kit v2 (Life technologies).

### qRT-PCR

One μg of total RNA was used to synthesize cDNA in a 20 μL reaction mixture using HiScript ^®^II Q RT SuperMix for qPCR (+gDNA wiper) (Vazyme, Nanjing, China) according to the manufacturer’s instructions. Real-time PCR was carried out on the ABI StepOnePlus^™^ Real-Time PCR systems. Gene-specific primer sequences of the reference and target genes are listed in Table 1 and were synthesized by Invitrogen Biotech Co. Ltd. (Shanghai, China). The following thermal profile was used for qRT-PCR: 95°C for 3 min, followed by 40 cycles of 95°C for 10 sec and 60°C for 30 sec. The relative gene expression was calculated using the 2^-ΔΔCT^ method. Both GAPDH and HPRT1 genes were used to normalize variations in the amount of starting material.

**Table 1:** Primer sequences used in quantitative real time PCR assays.

Gene	Accession No.	Primer, 5’-3’
*HSPA1B*	NM_005345.5	Sense: GCGAGGCGGACAAGAAGAA Antisense:GATGGGGTTACACACCTGCT
*HSPA9*	NM_004134.6	Sense: GGAAGGTAAACAAGCAAAGGTGC Antisense:CCAACAAGTCGCTCACCATCT
*HSP90AA1*	NM_005348.3	Sense: GCTTGACCAATGACTGGGAAG Antisense:AGCTCCTCACAGTTATCCATGA
*HSPD1*	NM_002156.4	Sense: CTACTGTACTGGCACGCTCTA Antisense:CAACAGCTAACATCACACCTCTC
*HSF1*	NM_005526.3	Sense: CCATGAAGCATGAGAATGAGGC Antisense:CTTGTTGACGACTTTCTGTTGC
*HSPB1*	NM_001540.4	Sense: TGGACCCCACCCAAGTTTC Antisense:CGGCAGTCTCATCGGATTTT
*METTL3*	NM_019852.4	Sense: CAAGCTGCACTTCAGACGAA Antisense: GCTTGGCGTGTGGTCTTT
*METTL14*	NM_020961.3	Sense: AGAAACTTGCAGGGCTTCCT Antisense: TCTTCTTCATATGGCAAATTTTCTT
*FTO*	NM_001080432.2	Sense: ACTTGGCTCCCTTATCTGACC Antisense: GTGCAGTGTGAGAAAGGCTT
*YTHDF2*	NM_001172828.1	Sense: CCTTAGGTGGAGCCATGATTG Antisense: TCTGTGCTACCCAACTTCAGT
*GAPDH*	NM_001289746.1	Sense: CGACCACTTTGTCAAGCTCA Antisense: AGGGGAGATTCAGTGTGGTG
*HPRT1*	NM_000194.2	Sense: TGACACTGGCAAAACAATGCA Antisense: GGTCCTTTTCACCAGCAAGCT

*HSPA1B* heat shock protein 70; *HSPA9* heat shock protein 70; *HSP90AA1* heat shock protein 90; *HSPD1* heat shock protein 60; *HSF1* heat shock factor 1; *HSPB1* heat shock protein 27; *METTL3* methyltransferase like 3; *METTL14* methyltransferase like 14; *FTO* fat mass and obesity associated; *YTHDF2* YTH domain family 2, *GAPDH* glyceraldehyde-3-phosphate dehydrogenase, *HPRT1* hypoxanthine phosphoribosyltransferase 1.

### Cell proliferation and viability assay

Cell proliferation was performed using the 3-(4,5-dimethylthiazol-2-yl)-2,5-diphenyltetrazolium bromide (MTT) assay according to the manufacturer’s instructions (Roche Applied Science). The transfected cells were plated in 96-well plates (3000 cells/well). Cell proliferation was determined at 24 h, 48 h, and 72 h after the transfection or heat shock, respectively.

### mRNA lifetime

METTL3 siRNA was transfected into HepG2 cells at 70–80% confluence in 24-well plates using Lipofectamine RNAiMAX (Invitrogen) following the manufacture’s protocols. After 48 h transfection, the cells were treated with actinomycin (5 μg/ml) for 6 h, 3 h, and 0 h before trypsinization and collection.

The total RNA was isolated using TRIzol. After reverse transcription, mRNA levels of transcripts of interest were detected by qRT-PCR. The degradation rate of RNA *k* was estimated by

$${\text{log}}_{2}\left(\frac{At}{A0}\right)=-kt$$

where *t* is transcription inhibition time (h), *A*_*t*_ and *A*_*0*_ represent mRNA quantity at time *t* and time *0*. Two *k* values were calculated: time 3 h versus time 0 h, and time 6 h versus time 0 h. The final lifetime was calculated by using the average of *k*_*3 h*_ *and k*_*6 h*_.

$$\text{t}\frac{1}{2}=\frac{2\text{ln}2}{k3h+k6\;h}$$

### Immunofluorescence staining

HepG2 cells grown on glass coverslips were fixed in 4% paraformaldehyde in phosphate-buffered saline (PBS) for 10 min at 4°C, and then were permeated with 0.1% Trition X-100 in PBS for 15 min. Dako blocking solution (Dakocytomation protein block serum-free) was used to block the nonspecific binding of antibodies for 30–60 min. The cells were then incubated with rabbit polyclonal anti-YTHDF2 (Proteintech, 24744-1-AP) diluted in Dakocytomation antibody diluents for 2 h at room temperature or overnight at 4°C followed by 1 h incubation at room temperature with the secondary Alexa series fluorescently labeled antibodies (1:1500 dilution). After washing with PBST for three times, prolong Gold antifade reagent with DAPI staining was used for nuclei detection (Life Technologies). Confocal microscopy images were captured by Leica SP5 II STED-CW Super-resolution Laser Scanning Confocal instrument and analysed by ImageJ software.

### Immunoblotting

Total cellular protein was isolated from HepG2 cells using Cell Lysis Buffer containing Complete Protease Inhibitor (Roche) and PMSF for determination of METTL3. The protein concentrations were determined using the BCA protein assay kit according to the protocol provided by the manufacturer (Nanjing Jiancheng Bioengineering Institue, Jiangsu, China). A total of 20 μg of protein with loading buffer was boiled for 5 min and electrophoretically resolved by 10% reducing SDS-PAGE gels. Protein was then transferred to nitrocellulose membranes. The membranes were blocked for 1 h in TBS containing 5% non-fat milk and 0.1% Tween-20, followed by incubation with mouse polyclonal anti-METTL3 (Novus, H00056339) or mouse monoclonal anti-GAPDH (Invitrogen, MA5-15738-HRP) antibodies overnight at 4°C. After incubation with horseradish-peroxidase-coupled secondary antibodies at room temperature for 1 h, immunoblots were visualized using enhanced chemiluminescence (ECL^Plus^)

### m^6^A-seq

For m^6^A immunoprecipitation, the procedure was modified from the previously reported methods [15]. In brief, total RNA was extracted using TRIzol reagent followed by purification using PolyATtract^®^ mRNA Isolation System III (Promega). Subsequently, purified mRNAs were digested using DNase I and then fragmented into roughly 100-nt fragments by incubation for 15 min at 70°C in fragmentation buffer (10 mM Tris-HCl, pH 7.0, 10 mM ZnCl_2_). 500 ng mRNA was saved as input control for RNA-seq. Five μg fragmented mRNA was incubated with 12 μg anti-m^6^A antibody (Synaptic Systems) in 1 × IP buffer (10 mM Tris-HCl, pH 7.4, 150 mM NaCl, and 0.1% Igepal CA-630) for 2 h at 4°C. At the same time, recombinant protein A bead (Invitrogen) was washed twice followed by incubation in 1 × IP buffer with 0.5 mg/ml BSA on a rotating wheel for 2 h at 4°C. The m^6^A-IP mixture was then incubated with protein A beads for additional 2 h at 4°C on a rotating wheel. After washing three times with IP buffer, bound mRNA was eluted using 100 μl elution buffer (6.7 mM *N*^6^-Methyladenosine-5’-monophosphate sodium salt in IP buffer) followed by ethanol and sodium acetate precipitation. Immunoprecipitated RNA fragments and comparable amounts of input were subjected to first-strand cDNA synthesis. Sequencing was performed on Illumina HiSeq2500 according to the manufacuture’s instructions.

### Statistical analysis

Comparisons between the mean ± SEM of two groups were calculated using Student’s unpaired two-tailed t test, performed with SPSS software. The following p values were considered to be statistically significant: p value ≤ 0.05 (*), p value ≤ 0.01 (**).

## Results

### Heat shock changes *HSP70*, *METTL3* and *YTHDF2* mRNA expression

**Fig 1:**
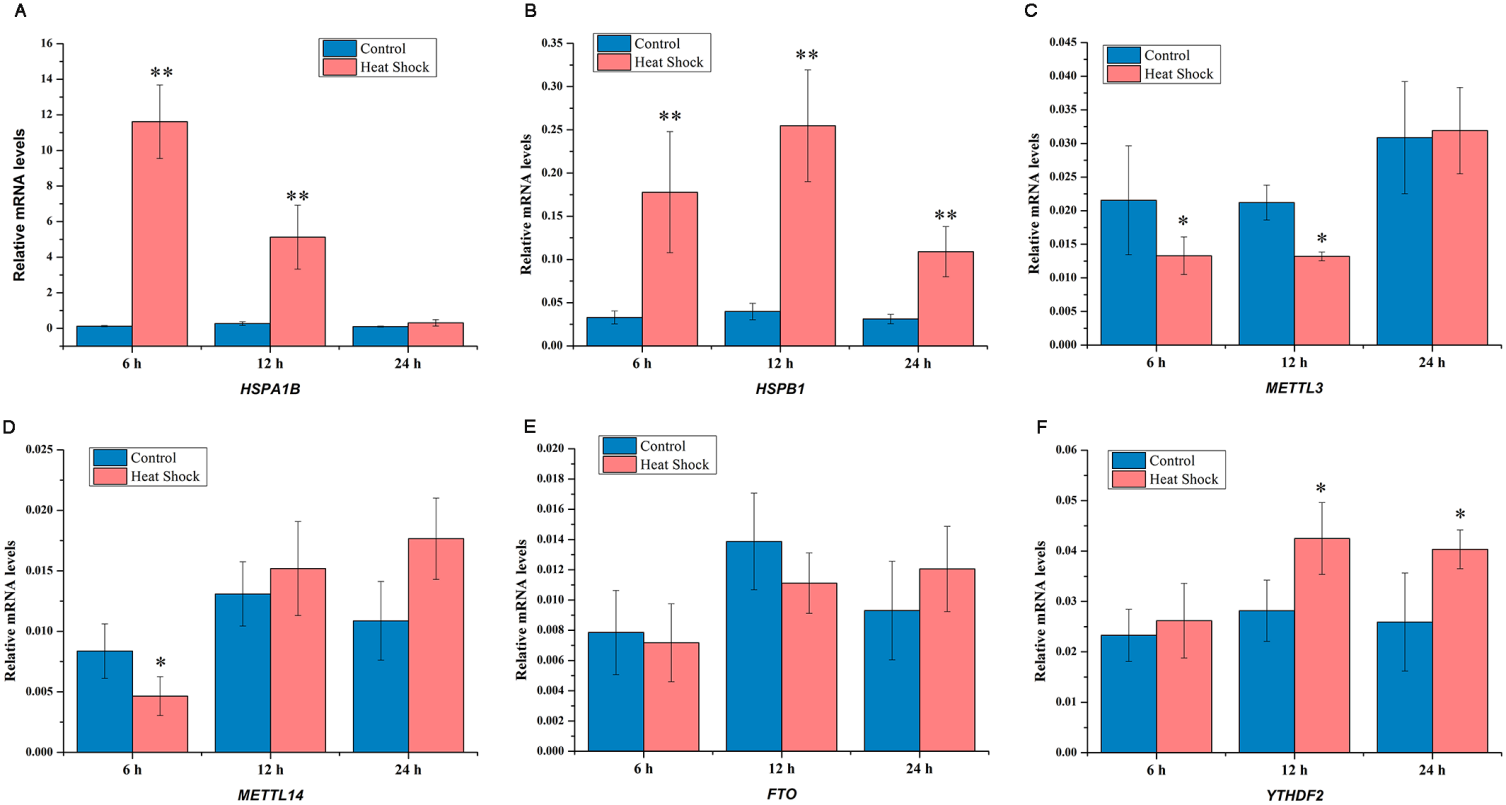
Effect of heat shock on *HSPs* and m^6^A mRNA methylation related genes. Expression of *HSPA1B* (*HSP70*) (**A**), *HSPB1* (*HSP27*) (**B**), *METTL3* (**C**), *METTL14* (**D**), *FTO* (**E**), and *YTHDF2* (**F**) mRNA at 6 h, 12 h, 24 h after heat shock in HepG2 cells. Data are shown as mean ± SEM (n = 3). *p value ≤ 0.05, **p value ≤ 0.01.

Compared to control group, the expression of *HSP70* mRNA was increased at 6 h, 12 h, and 24 h after heat shock stress in HepG2 cells (*p* < 0.05) (Fig 1A and 1B). Decrease of *METTL3* mRNA expression was observed at 6 h and 12 h after heat shock stress compared to control (*p* < 0.05) (Fig 1C). Heat shock inhibited the expression of *METTL14* mRNA at 6 h after heat treatment (*p* < 0.05) (Fig 1D). We found that there was no change in *FTO* expression after heat shock stress (Fig 1E). However, the expression of *YTHDF2* mRNA was enhanced at 12 h and 24 h after heat shock stress (*p* < 0.05) (Fig 1F).

### Heat shock changes localization of YTHDF2

**Fig 2:**
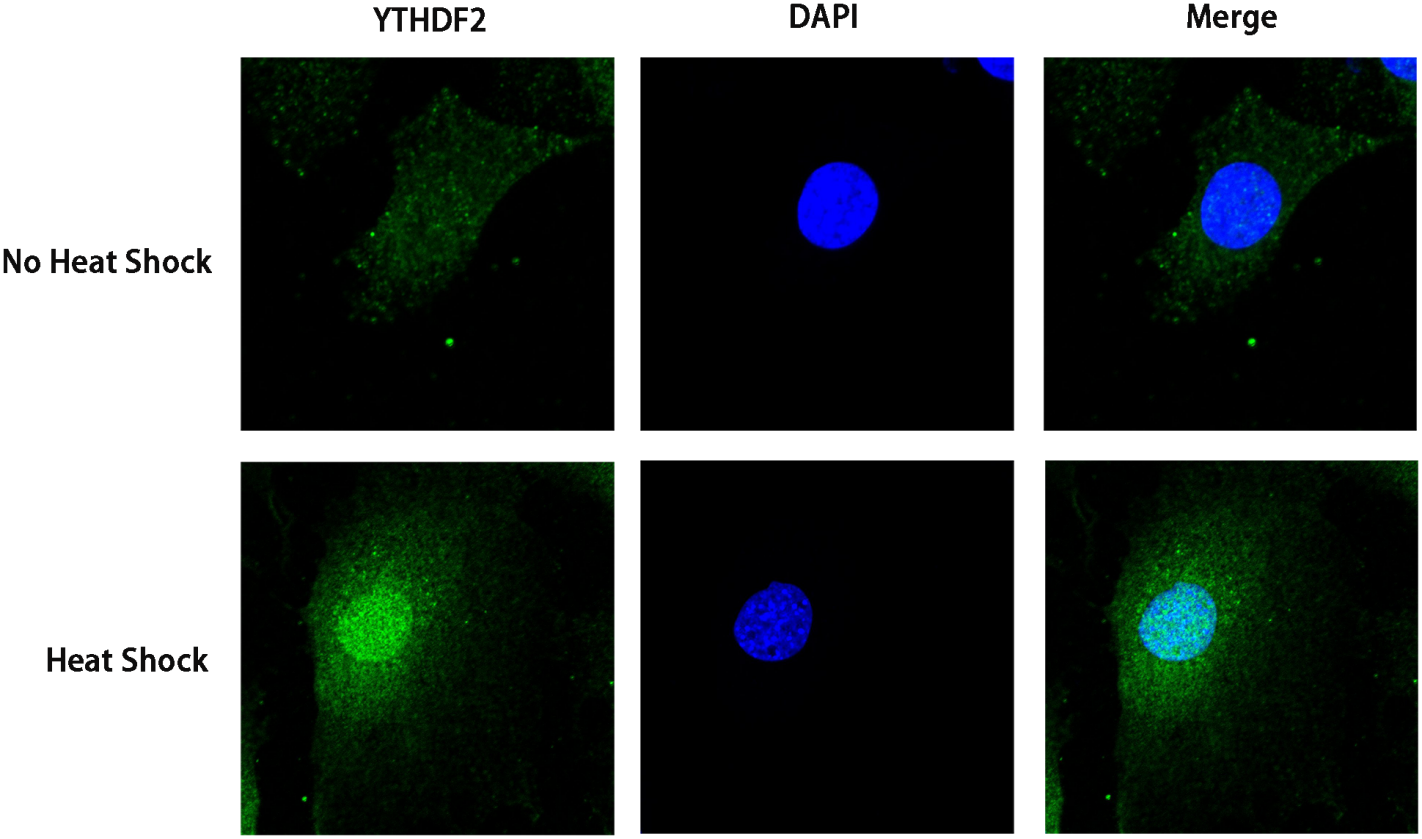
Localization of YTHDF2 under heat shock. The majority of YTHDF2 resided in the cytosol in normal conditions, whereas nearly all YTHDF2 translocated into the nucleus from the cytosol under heat shock stress. *Scale bar* = 88 μm.

Surprisingly, we found that the majority of YTHDF2 resided in the cytosol under normal condition, however, under heat shock stress nearly all YTHDF2 translocated into the nucleus from the cytosol (Fig 2).

### METTL3 knockdown affects HSPs expression and cell proliferation

**Fig 3:**
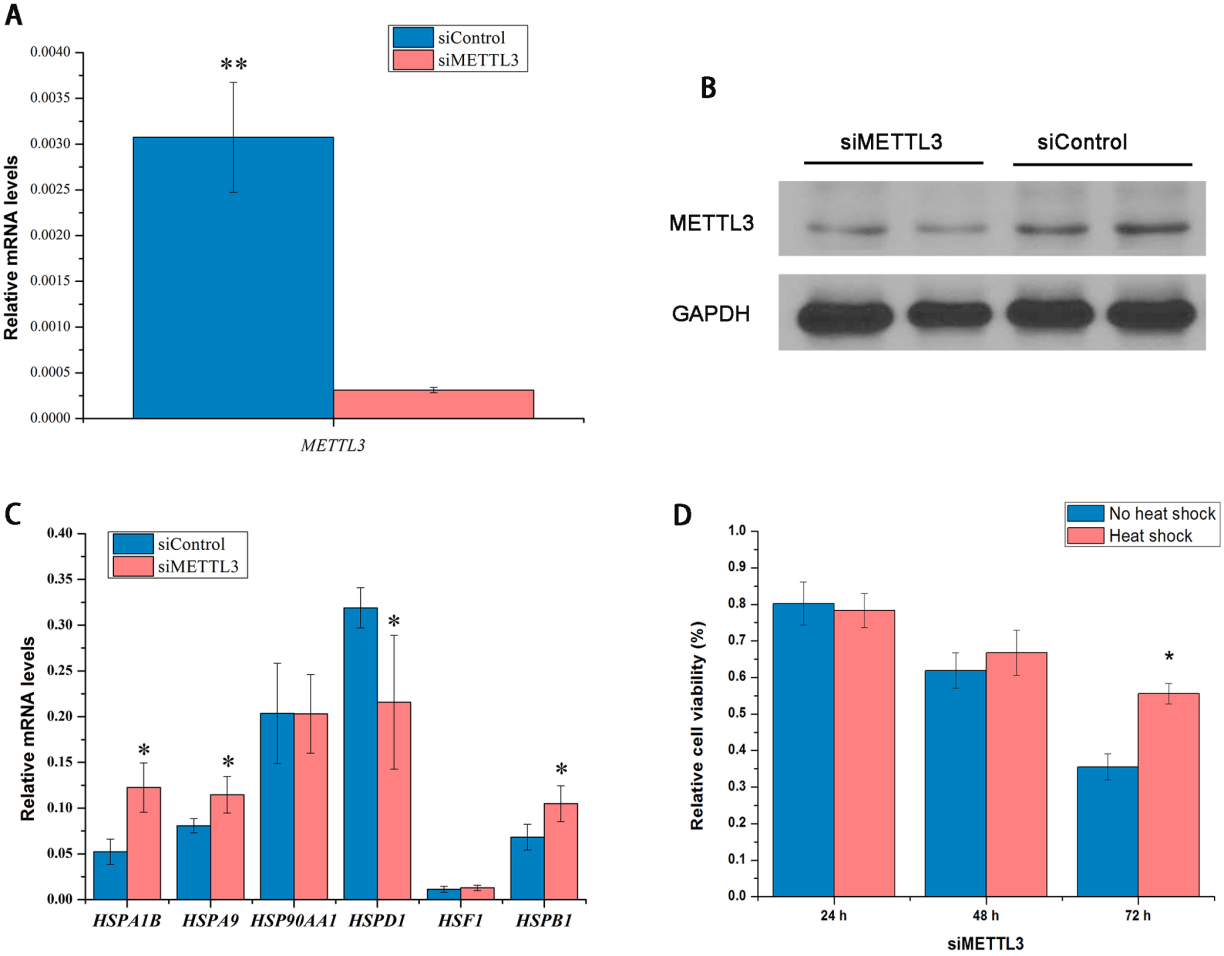
Effect of METTL3 knockdown on HSPs and cell viability in HepG2 cells. Expression of *METTL3* mRNA and protein in HepG2 cells after METTL3 knockdown (**A** and **B**). (n = 3). Expression of *HSPA1B* (*HSP70*), *HSPA9* (*HSP70*), *HSP90AA1* (*HSP90*), *HSPD1* (*HSP60*), *HSF1*, and *HSPB1* (*HSP27*) mRNA upon METTL3 knockdown in HepG2 cells **(C)** (n = 3). The relative cell viability determined by MTT at 24, 48, and 72 h post-transfection of METTL3 siRNA with or without heat shock pretreatment (**D**) (n = 6). Data are shown as mean ± SEM. *p value ≤ 0.05, **p value ≤ 0.01.

In order to determine the role of m^6^A RNA methylation on the regulation of HSPs, *METTL3* was knockdowned using siRNA. Although METTL14 shows a similar regulation as METTL3, here we only observed *METTL3* knockdown because *METTL3* and *METTL14* form a stable heterodimer core complex of METTL3-METTL14 that functions in cellular m^6^A deposition on mammalian nuclear RNAs and the phenotypes of *METTL14* knockdown are the same with *METTL3*. In addition, it has been very well-described that METTL3 is m^6^A specific methyltransferases and regulates the levels of m^6^A [11]. Knockdown of METTL3 significantly decreased m^6^A methylation in many type of cell line and in vivo. Here, we did not confirm this. Our results shows that *METTL3* mRNA was decreased by 89% at 48 h after METTL3 knockdown in HepG2 cells (*p* < 0.05) (Fig 3A). The levels of METTL3 protein were also significantly decreased in HepG2 cells (Fig 3B). METTL3 knockdown in HepG2 cells significantly increased the expression of *HSP70* and *HSP27* mRNA compared to control (*p* < 0.05) (Fig 3C). In contrast, the levels of *HSP60* mRNA were inhibited following siMETTL3 treatment compared to the control (*p* < 0.05) (Fig 3C). There were no differences in abundance of *HSP90* and *HSF1* mRNA between control and siMETTL3 groups. In addition, knockdown of *METTL3* using a specific siRNA led to the decrease of relative cell viability in HepG2 cells determined by MTT (3-[4,5-dimethylthiazol-2-yl]-2, 5-diphenyl-tetrazolium bromide) at 24, 48, and 72 h after transfection without heat shock (Fig 3D). However, heat shock pretreatment attenuated the decrease of relative cell viability induced by knockdown of *METTL3* in HepG2 cells at 72 h (Fig 3D).

### Knockdown of METTL3 does not change *HSPA1B* lifetime

**Fig 4:**
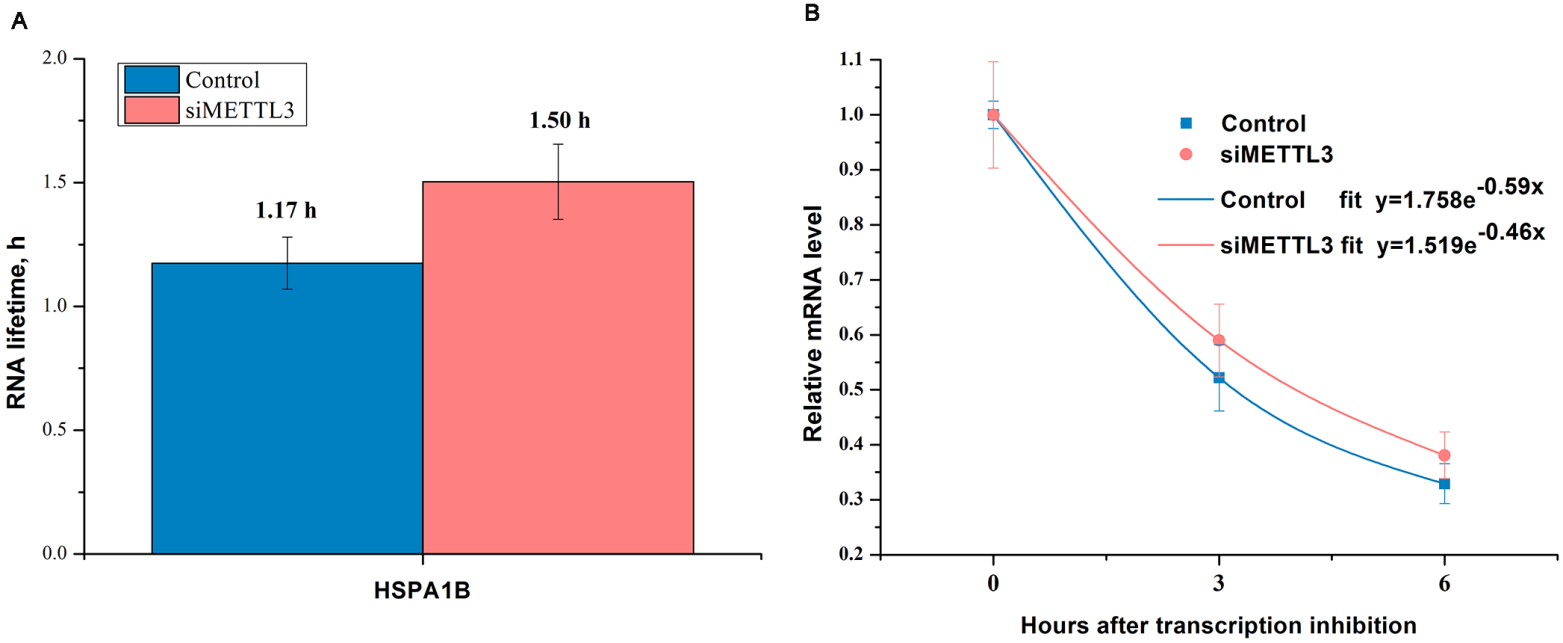
Effect of METTL3 knockdown on the lifetime of HSPA1B in HepG2 cells. Lifetime of *HSPA1B* (*HSP70*) mRNA in the samples following knockdown of *METTL3* in HepG2 cells (**A**). The relative mRNA levels of *HSPA1B* (*HSP70*) in the samples following knockdown of *METTL3* in HepG2 cells at 0 h, 3 h, and 6 h (**B**).

The results of *HSPA1B* lifetime is shown in Fig 4A and 4B. The results showed that METTL3 knockdown in HepG2 cells did not affect the lifetime of *HSPA1B* compared to control.

### YTHDF2 knockdown affects HSPs expression and cell proliferation

**Fig 5:**
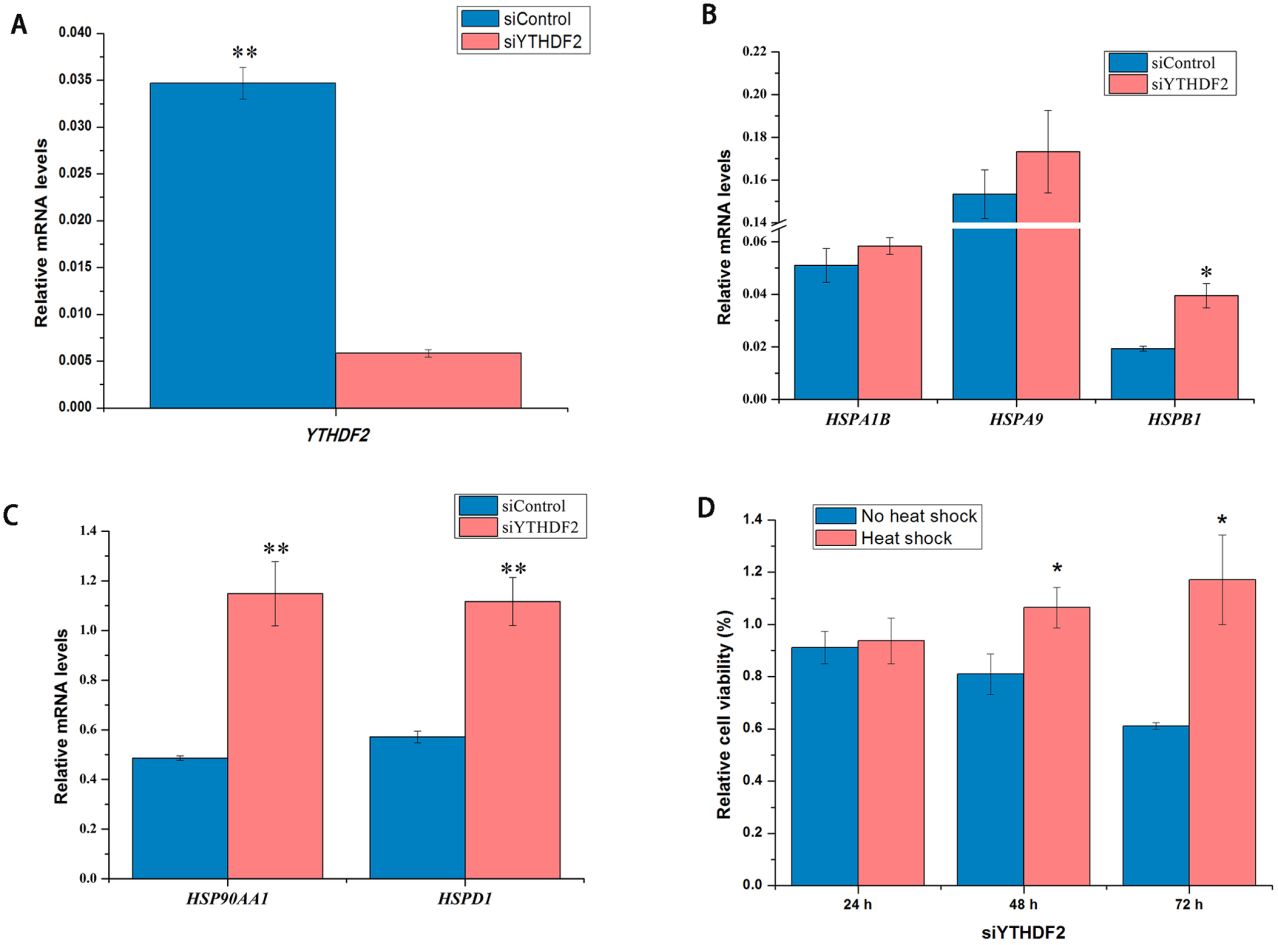
Effect of YTHDF2 on HSPs mRNA expression and cell viability in HepG2 cells. YTHDF2 knockdown decreased YTHDF2 mRNA in HepG2 cells (**A**). Expression of *HSPA1B* (*HSP70*), *HSPA9* (*HSP70*), *HSPB1* (*HSP27*), *HSP90AA1* (*HSP90*), *HSPD1* (*HSP60*) mRNA from the sample of YTHDF2 knockdown in HepG2 cells (**B** and **C**). The relative cell viability determined by MTT at 24, 48, and 72 h after knockdown of YTHDF2 with or without heat shock pretreatment (**D**) (n = 6). Data are shown as mean ± SEM. *p value ≤ 0.05, **p value ≤ 0.01.

In order to illuminate the function of m^6^A binding protein, YTHDF2 was knockdowned in HepG2 cells. *YTHDF2* mRNA was decreased by 83% at 48 h after YTHDF2 knockdown in HepG2 cells (*p* < 0.05) (Fig 5A). Because YTHDF2 siRNA has been validated very well by our collaborator Dr. Chuan He, we did not further confirm the knockdown YTHDF2 using Western blot. The results showed that differences in *HSPA1B* and *HSPA9* mRNA expression were not observed between control and siYTHDF2 samples (Fig 5B). However, expression of *HSP90AA1*, *HSPD1*, and *HSPB1* mRNA were significantly increased by knockdown of YTHDF2 in HepG2 cells (Fig 5B and 5C). Furthermore, *YTHDF2* knockdown reduced the relative cell viability in HepG2 cells at 24, 48, and 72 h after transfection without heat shock (Fig 5D). Surprisingly, heat shock pretreatment increased the relative cell viability after knockdown of *YTHDF2* (Fig 5D).

### Profile of HSPs gene-m^6^A methylation

**Fig 6:**
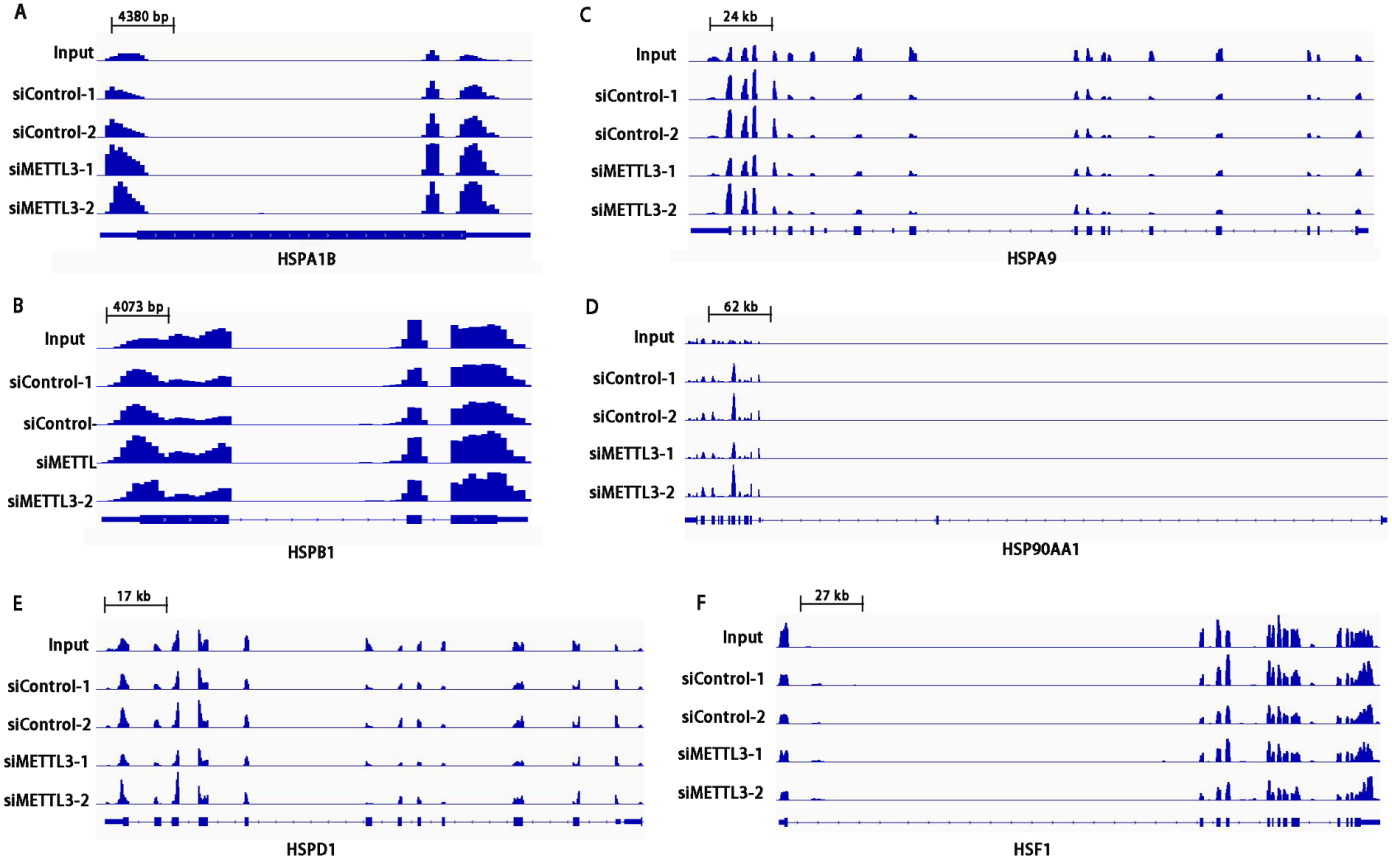
M^6^A methylated peaks of HSPs mRNA. Integrative genomics viewer (IGV) plots showing m^6^A methylated peaks for *HSPA1B* (*HSP70*) (**A**), *HSPB1* (*HSP27*) (**B**), *HSPA9* (*HSP70*) (**C**), *HSP90AA1* (*HSP90*) (**D**), *HSPD1* (*HSP60*) (**E**), *HSF1* (**F**) mRNA in HepG2 cells. Blue boxes represent exons and blue lines represent introns. *n* = 2.

Using previously described methods for m^6^A-seq [22], we sequenced the entire methylated RNA species purified from HepG2 cells. Coinciding with the previously reported pattern of m^6^A peaks [11, 13], the m^6^A sites of *HSPA1B*, *HSPB1*, *HSPA9*, *HSP90AA1*, *HSPD1*, *HSF1* transcripts mainly distribute on exons and around stop codons (Fig 6A–6F). In addition, *HSPA1B*, *HSPB1*, *HSPA9*, and *HSPD1* transcripts were found to have m^6^A enrichments in both the 5’UTR and 3’UTR. M^6^A enrichment for *HSP90AA1* transcript was found only in the 5’UTR (Fig 6D). In contrast, HSF1 transcript showed m^6^A enrichments mainly in the 3’UTR (Fig 6F). Moreover, knockdown of *METTL3* changed methylation patterns of *HSPA1B*, *HSPA9*, *HSPB*1, and *HSPD1*.

## Discussion

As one of the most prevalent modifications in mRNAs, m^6^A methylation has emerged as a key post-transcriptional regulator of gene expression [23]. In the present study, we observed that METTL3 or YTHDF2 knockdown significantly changed HSPs gene expression, and heat shock induced nuclear localization of YTHDF2. M^6^A methylation peaks of HSPs are mainly enriched on exons and around stop codons. The precise mechanism for different expression of HSPs induced by m^6^A RNA methylation requires further investigation.

As a novel epitranscriptomic marker, m^6^A is a widespread modification present in over 7,000 human genes, including circadian genes [24], *p53* [15], *Notch1α* [25], and *XIST* [26] genes. Zhou et al. [21] also showed that although there was no effect on *HSP70* mRNA expression, YTHDF2 activated its mRNA translation under heat shock stress. However, the effect of m^6^A RNA methylation on other stress-inducible chaperone HSP90, HSP60, and HSP27 is still unknown. Here we found that heat shock treatment significantly increased *HSP70* mRNA with a decrease in METTL3, whereas we observed an increase in YTHDF2, suggesting a dynamic m^6^A mRNA methylation response to environmental changes. In order to investigate whether these changes are associated with HSPs gene expression, we inhibited METTL3 in HepG2 cells using siRNA. We found that *HSPA1B*, *HSPA9*, and *HSP60* mRNA showed significant changes, but no differences were observed for *HSP90AA1* and *HSF1* mRNA. These different effects may be associated with different m^6^A site and abundance on HSPs transcript. Since m^6^A RNA methylation contributes to mRNA stability, we performed HSP70 lifetime analysis in HepG2 cells and found that METTL3 knockdown did not change HSP70 mRNA lifetime, suggesting that it may have other regulatory mechanisms of m^6^A on HSPs.

M^6^A is recognized by the YTH domain family proteins [27]. There are five proteins (YTHDF1-3, YTHDC1, and YTHDC2) that contain the YTH domain in human cells. YTHDF1-3 are primarily cytoplasmic, whereas YTHDC1 is located primarily in the nucleus [13, 27]. YTHDF1 binds m^6^A-modified mRNAs through interactions with initiation factors and ribosomes to increase translational output [28]. YTHDF2 regulates mRNA decay by binding to m^6^A transcripts, which results in their re-localization from the pool of ribosome-associated translatable transcripts to cellular RNA decay sites such as P-bodies [13]. In the present study, YTHDF2 knockdown did not change *HSPA1B*, *HSPA9* mRNA expression, but significantly increased *HSP90AA1*, *HSPD1*, and *HSPB1* mRNA expression. Altered expression of HSPs may be associated with YTHDF2 function, which depends on the m^6^A site of the target gene. Immunofluorescence staining showed that the majority of YTHDF2 was present in the cytosol, however, nearly all YTHDF2 translocated from the cytosol into the nucleus under heat shock, further confirming that YTHDF2 regulates HSPs mRNA expression.

Different sites and abundance of m^6^A on transcript may lead to altered gene output. In addition, the function of YTHDF2 may depend on the cellular context and the m^6^A site of the target gene. A previous study showed that YTHDF2 knockdown increased the m^6^A/A ratio and the stabilization of the RNA targets [13]. However, upon heat shock stress, the nuclear YTHDF2 protects the 5’UTR of stress-induced transcripts from demethylation and activates translation [21]. Using m^6^A-seq, we dissected the profile of HSPs RNA methylation and found that there are different distribution characteristic of m^6^A between HSPs transcript. We also observed changes of methylation patterns of HSPs mRNA after *METTL3* knockdown. These results, to some extent, suggest that different expression of *HSP70*, *HPS90*, *HSP60*, and *HSP27* are modulated by METTL3 or YTHDF2 knockdown in HepG2 cells *in vitro*.

M^6^A modification on gene expression impacts a variety of physiological events, including mammalian embryonic stem cell fate [29], X chromosome inactivation [26], zebrafish maternal-to-zygotic transition [16], haematopoietic stem and progenitor cell specification [25], metabolic diseases, and the stability of the mammalian circadian clock [24]. In the present study, we also observed that decrease of m^6^A RNA methylation reduces cell viability in HepG2 cells. However, heat shock pretreatment attenuated the decrease of cell viability induced by knockdown of *METTL3* or *YTHDF2* in HepG2 cells. Further investigation is needed to reveal the underlying mechanism of these changes.

In conclusion, the expression of HSPs is modulated by m^6^A RNA methylation. This finding would provide insights into the new regulatory mechanisms of HSPs in normal and stress situations.

## References

[1] Oellmy R, Boellmann F. Chaperone regulation of the heat shock protein response Molecular Aspects of the Stress Response: Chaperones, Membranes and Networks: Springer; 2007 p. 89–99. 10.1007/978-0-387-39975-1_9 17205678

[2] Zhong X, Li W, Huang X, Zhang L, Yimamu M, Raiput N, et al Impairment of cellular immunity is associated with overexpression of heat shock protein 70 in neonatal pigs with intrauterine growth retardation. Cell Stress Chaperones. 2012;17(4):495–505. doi: 10.1007/s12192-012-0326-6 22270614 10.1007/s12192-012-0326-6 PMC3368032

[3] Zhong X, Wang T, Zhang X, Li W. Heat shock protein 70 is upregulated in the intestine of intrauterine growth retardation piglets. Cell Stress Chaperones. 2010;15(3):335–42. doi: 10.1007/s12192-009-0148-3 19830596 10.1007/s12192-009-0148-3 PMC2866992

[4] Kampinga HH, Hageman J, Vos MJ, Kubota H, Tanguay RM, Bruford EA, et al Guidelines for the nomenclature of the human heat shock proteins. Cell Stress Chaperones. 2009;14(1):105–11. doi: 10.1007/s12192-008-0068-7 18663603 10.1007/s12192-008-0068-7 PMC2673902

[5] Ciocca DR, Arrigo AP, Calderwood SK. Heat shock proteins and heat shock factor 1 in carcinogenesis and tumor development: an update. Arch Toxicol. 2013;87(1):19–48. doi: 10.1007/s00204-012-0918-z 22885793 10.1007/s00204-012-0918-z PMC3905791

[6] Smith ST, Petruk S, Sedkov Y, Cho E, Tillib S, Canaani E, et al Modulation of heat shock gene expression by the TAC1 chromatin-modifying complex. Nat Cell Biol. 2004;6(2):162 doi: 10.1038/ncb1088 14730313 10.1038/ncb1088

[7] Miozzo F, Sabéran-Djoneidi D, Mezger V. HSFs, stress sensors and sculptors of transcription compartments and epigenetic landscapes. J Mol Biol. 2015;427(24):3793–816. doi: 10.1016/j.jmb.2015.10.007 26482101 10.1016/j.jmb.2015.10.007

[8] Marinova Z, Leng Y, Leeds P, Chuang D-M. Histone deacetylase inhibition alters histone methylation associated with heat shock protein 70 promoter modifications in astrocytes and neurons. Neuropharmacology. 2011;60(7):1109–15. 20888352 10.1016/j.neuropharm.2010.09.022 PMC3036778

[9] Fritah S, Col E, Boyault C, Govin J, Sadoul K, Chiocca S, et al Heat-shock factor 1 controls genome-wide acetylation in heat-shocked cells. Mol Biol Cell. 2009;20(23):4976–84. doi: 10.1091/mbc.E09-04-0295 19793920 10.1091/mbc.E09-04-0295 PMC2785740

[10] Wei C-M, Gershowitz A, Moss B. Methylated nucleotides block 5′ terminus of HeLa cell messenger RNA. Cell. 1975;4(4):379–86. 164293 10.1016/0092-8674(75)90158-0

[11] Liu J, Yue Y, Han D, Wang X, Fu Y, Zhang L, et al A METTL3-METTL14 complex mediates mammalian nuclear RNA N^6^-adenosine methylation. Nat Chem Biol. 2014;10(2):93–5. doi: 10.1038/nchembio.1432 24316715 10.1038/nchembio.1432 PMC3911877

[12] Jia G, Fu Y, Zhao X, Dai Q, Zheng G, Yang Y, et al N^6^-methyladenosine in nuclear RNA is a major substrate of the obesity-associated FTO. Nat Chem Biol. 2011;7(12):885–7. doi: 10.1038/nchembio.687 22002720 10.1038/nchembio.687 PMC3218240

[13] Wang X, Lu Z, Gomez A, Hon GC, Yue Y, Han D, et al N^6^-methyladenosine-dependent regulation of messenger RNA stability. Nature. 2014;505(7481):117–20. doi: 10.1038/nature12730 24284625 10.1038/nature12730 PMC3877715

[14] Wang X, He C. Reading RNA methylation codes through methyl-specific binding proteins. RNA Biol. 2014;11(6):669–72. doi: 10.4161/rna.28829 24823649 10.4161/rna.28829 PMC4156498

[15] Dominissini D, Moshitch-Moshkovitz S, Schwartz S, Salmon-Divon M, Ungar L, Osenberg S, et al Topology of the human and mouse m^6^A RNA methylomes revealed by m^6^A-seq. Nature. 2012;485(7397):201–6. doi: 10.1038/nature11112 22575960 10.1038/nature11112

[16] Zhao BS, Wang X, Beadell AV, Lu Z, Shi H, Kuuspalu A, et al m^6^A-dependent maternal mRNA clearance facilitates zebrafish maternal-to-zygotic transition. Nature. 2017;542(7642):475 doi: 10.1038/nature21355 28192787 10.1038/nature21355 PMC5323276

[17] Xiao W, Adhikari S, Dahal U, Chen Y-S, Hao Y-J, Sun B-F, et al Nuclear m 6 A reader YTHDC1 regulates mRNA splicing. Mol Cell. 2016;61(4):507–19. 26876937 10.1016/j.molcel.2016.01.012

[18] Zheng G, Dahl JA, Niu Y, Fedorcsak P, Huang C-M, Li CJ, et al ALKBH5 is a mammalian RNA demethylase that impacts RNA metabolism and mouse fertility. Mol Cell. 2013;49(1):18–29. doi: 10.1016/j.molcel.2012.10.015 23177736 10.1016/j.molcel.2012.10.015 PMC3646334

[19] Meyer KD, Patil DP, Zhou J, Zinoviev A, Skabkin MA, Elemento O, et al 5′ UTR m^6^A promotes cap-independent translation. Cell. 2015;163(4):999–1010. doi: 10.1016/j.cell.2015.10.012 26593424 10.1016/j.cell.2015.10.012 PMC4695625

[20] Xiang Y, Laurent B, Hsu C-H, Nachtergaele S, Lu Z, Sheng W, et al RNA m^6^A methylation regulates the ultraviolet-induced DNA damage response. Nature. 2017;543:573–6. doi: 10.1038/nature21671 28297716 10.1038/nature21671 PMC5490984

[21] Zhou J, Wan J, Gao X, Zhang X, Qian S-B. Dynamic m^6^A mRNA methylation directs translational control of heat shock response. Nature. 2015;526(7574):591 doi: 10.1038/nature15377 26458103 10.1038/nature15377 PMC4851248

[22] Dominissini D, Moshitch-Moshkovitz S, Salmon-Divon M, Amariglio N, Rechavi G. Transcriptome-wide mapping of N^6^-methyladenosine by m^6^A-seq based on immunocapturing and massively parallel sequencing. Nat Protoc. 2013;8(1):176–89. doi: 10.1038/nprot.2012.148 23288318 10.1038/nprot.2012.148

[23] Fu Y, Dominissini D, Rechavi G, He C. Gene expression regulation mediated through reversible m^6^A RNA methylation. Nature reviews Genetics. 2014;15(5):293 doi: 10.1038/nrg3724 24662220 10.1038/nrg3724

[24] Fustin J-M, Doi M, Yamaguchi Y, Hida H, Nishimura S, Yoshida M, et al RNA-methylation-dependent RNA processing controls the speed of the circadian clock. Cell. 2013;155(4):793–806. doi: 10.1016/j.cell.2013.10.026 24209618 10.1016/j.cell.2013.10.026

[25] Zhang C, Chen Y, Sun B, Wang L, Yang Y, Ma D, et al m^6^A modulates haematopoietic stem and progenitor cell specification. Nature. 2017;549(7671):273–6. doi: 10.1038/nature23883 28869969 10.1038/nature23883

[26] Patil DP, Chen C-K, Pickering BF, Chow A, Jackson C, Guttman M, et al m^6^A RNA methylation promotes XIST-mediated transcriptional repression. Nature. 2016;537(7620):369 doi: 10.1038/nature19342 27602518 10.1038/nature19342 PMC5509218

[27] Xu C, Wang X, Liu K, Roundtree IA, Tempel W, Li Y, et al Structural basis for selective binding of m^6^A RNA by the YTHDC1 YTH domain. Nat Chem Biol. 2014;10(11):927–9. doi: 10.1038/nchembio.1654 25242552 10.1038/nchembio.1654

[28] Wang X, Zhao BS, Roundtree IA, Lu Z, Han D, Ma H, et al N^6^-methyladenosine modulates messenger RNA translation efficiency. Cell. 2015;161(6):1388–99. doi: 10.1016/j.cell.2015.05.014 26046440 10.1016/j.cell.2015.05.014 PMC4825696

[29] Batista PJ, Molinie B, Wang J, Qu K, Zhang J, Li L, et al m^6^A RNA modification controls cell fate transition in mammalian embryonic stem cells. Cell stem cell. 2014;15(6):707–19. doi: 10.1016/j.stem.2014.09.019 25456834 10.1016/j.stem.2014.09.019 PMC4278749

